# Beyond Neurodegeneration: White Matter Vacuolation as a Primary Myelin Defect

**DOI:** 10.3390/ijms27157066

**Published:** 2026-08-06

**Authors:** Sneha Misra, Teresa M. Gunn

**Affiliations:** 1 Touro College of Osteopathic Medicine–Montana, Great Falls, MT 59405, USA; smisra@student.touro.edu; 2 Weissman Hood Institute at Touro University (McLaughlin Research Institute), Great Falls, MT 59405, USA

**Keywords:** spongiform encephalopathy, CNS vacuolation, status spongiosis, myelination, myelinopathy, leukodystrophies

## Abstract

Spongiform degeneration, or status spongiosis, is characterized by vacuoles within the central nervous system. It appears in numerous neurological diseases, including transmissible spongiform encephalopathies, mitochondrial disorders, and lysosomal storage diseases. Traditionally considered secondary to neurodegeneration, vacuolar changes frequently involve white matter and form within the myelin sheath. This review examines the evidence from various diseases and genetic models that exhibit this pathology to support the hypothesis that white matter vacuolation represents a myelin defect and explores potential causative mechanisms. Our findings suggest that spongiform change in white matter represents a common endpoint of pathway disruptions that lead to metabolic or ionic dyshomeostasis, causing an osmotic imbalance and vacuole formation within myelin. We advocate for further research into myelin-preserving pathways as potential therapeutic avenues to treat conditions exhibiting this pathology.

## 1. Introduction

**Figure 1:**
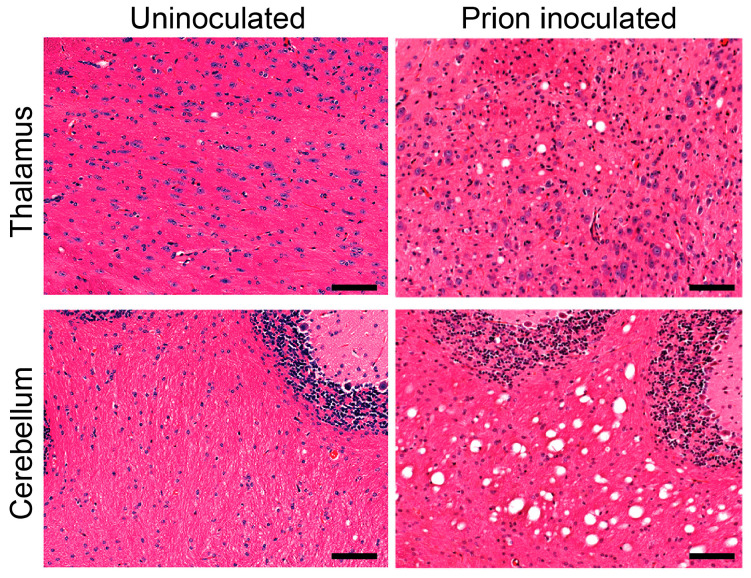
Brightfield images of hematoxylin and eosin-stained paraffin sections of thalamus (**top**) and cerebellum (**bottom**) of uninoculated mice (unaffected) and mice inoculated with Rocky Mountain Laboratory (RML) prions (with spongiform encephalopathy). Scale bars: 100 microns. Modified and reproduced from Silvius et al., PLOS ONE, 2013 [2] under the terms of the Creative Commons Attribution License (CC BY).

White matter vacuolation, also referred to as spongiform change or vacuolar degeneration, is a distinctive histopathological feature characterized by the formation of fluid-filled spaces within the myelinated nerve tracts of the central nervous system (CNS) (Figure 1). While spongiform pathology is best known for its association with prion disease, it is not unique to a single disorder; rather, it represents a shared pathological hallmark across a broad range of toxic, metabolic, genetic, and neurodegenerative conditions, many of which are discussed in this review. Traditionally, the significance of white matter vacuolation has been unclear and it was often dismissed as either a post-mortem artifact or a byproduct of neuronal or glial cell death. However, the frequent localization of vacuolar changes to white matter-rich regions of the CNS suggests the possibility of a direct link to abnormalities in myelin biology.

This review re-evaluates white matter vacuolation through the framework of modern myelin biology, exploring the hypothesis that this pathology reflects a primary manifestation of intrinsic defects in the metabolic and structural processes required to maintain homeostasis of the myelin–axon interface. In order to investigate the mechanistic basis of CNS vacuolation, we performed comprehensive searches using PubMed and the Mouse Genome Database [1] to identify relevant human clinical conditions and murine genetic models. This review synthesizes evidence across the spectrum of diseases associated with white matter vacuolation and highlights converging pathways that drive vacuolar change specifically within myelin-rich white matter tracts of the CNS.

## 2. Myelination

**Figure 2:**
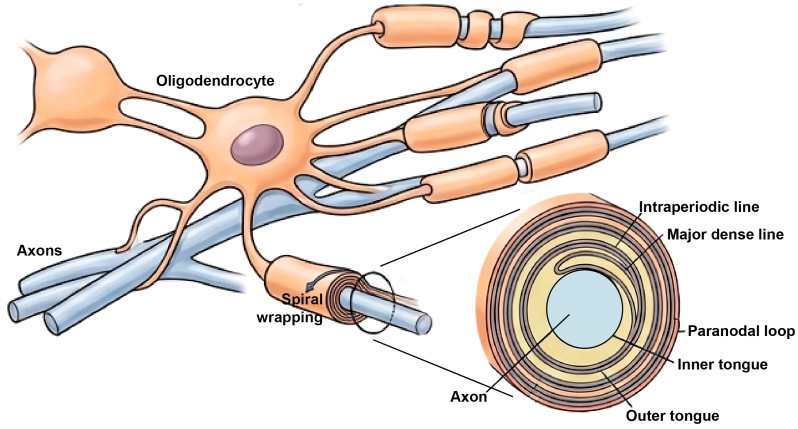
CNS Myelination. In the CNS, one oligodendrocyte can myelinate multiple axons. Ensheathment involves “inside-out” spiral wrapping of the oligodendrocyte plasma membrane around axons, forming a multi-layered stack of alternating protein and lipid layers. Viewed in cross-section, the intraperiodic line, representing the tightly apposed outer (extracellular) faces of the spiraled plasma membrane alternates with the major dense line, which represents the fused inner (cytoplasmic) faces of the membrane. The inner tongue is the innermost layer of the myelin sheath, situated directly between the compacted myelin and the axon’s membrane, and represents the active “growing front” of the myelin sheath. The outer tongue is the outermost layer of the myelin sheath that remains continuous with the oligodendrocyte cell body and provides the metabolic support necessary to maintain the health and electrical insulation of the nerve.

Myelination is the process by which neuronal axons are wrapped in a lipid-rich membrane (the myelin sheath), which insulates axons to facilitate rapid action potential propagation and provides metabolic support (Figure 2) [3]. Myelin is produced by mature oligodendrocytes in the CNS and by Schwann cells in the peripheral nervous system (PNS). While each Schwann cell myelinates a single axon, individual oligodendrocytes in the CNS can ensheathe one or more axons. Myelination involves “inside-out” spiral wrapping of the oligodendrocyte plasma membrane around axons, forming a multi-layered stack of alternating protein and lipid layers (Figure 2) [4]. It is a tightly regulated process that involves bidirectional communication between axons and oligodendroglia and is typically dependent on axonal activity. As discussed in detail below, oligodendrocytes in the CNS generate and maintain myelin through intricate intercellular communication, lipid synthesis, and precise regulation of metal ions like iron. Disruptions in any of these processes can destabilize myelin and impair remyelination.

Ultra-structurally, white matter vacuoles most commonly stem from spongiform myelinopathy, where intramyelinic edema, splitting of the myelin lamellae at the intraperiod line, or swelling of oligodendroglial processes results in a characteristic “spongy” appearance of the tissue when examined microscopically. This disruption of the myelin sheath architecture can severely compromise axonal conduction velocity, resulting in progressive motor deficits, sensory loss, and global cognitive dysfunction, but in some cases it may present with mild or no symptoms, or even be reversible.

## 3. Spongiform Degeneration in Transmissible Spongiform Encephalopathies (TSEs)

Transmissible spongiform encephalopathies (TSEs) such as Creutzfeldt-Jakob disease (CJD) in humans, scrapie in sheep, and bovine spongiform encephalopathy (BSE, or “mad cow disease”) in cattle, are a group of fatal neurodegenerative disorders caused by the accumulation of the misfolded, pathogenic form (scrapie, PrP^Sc^) of the prion protein [5,6]. A hallmark feature of TSE pathology is spongiform degeneration, in which microscopic vacuoles appear within neural tissues, giving the brain a sponge-like appearance [7,8]. While gray matter vacuolation, particularly in the neuropil, is prominent in TSEs, numerous studies have also documented significant changes in white matter [9,10,11]. Electron microscopic examination of vacuoles found within the myelin sheath revealed splitting of the myelin lamellae and formation of intramyelinic vacuoles in affected regions [8,12,13]. White matter pathology in TSEs also encompasses disruption of myelin architecture, accumulation of abnormal protein aggregates within myelin, and degenerative changes in oligodendrocytes [11,14]. Collectively, these observations suggest that myelin damage may be a central feature of TSE pathology rather than it being a downstream consequence of neuronal death.

### 3.1. Aberrant Myelination Associated with Loss of PrP^C^

Under physiological conditions, normal cellular prion protein (PrP^C^) is predominantly expressed in neurons, but it is also found in glial cells, including oligodendrocytes [15,16]. Several lines of evidence indicate that PrP^C^ plays an important role in myelin health and maintenance. *Prnp* knockout (*Prnp*^−/−^) mice, which lack the gene encoding PrP^C^, initially developed normal peripheral and central myelin. However, as these animals aged, they exhibited progressive white matter vacuolation, reactive astrogliosis, and deficits in motor coordination and balance [17]. Peripheral myelin pathology in *Prnp*^−/−^ mice could be rescued by neuron-specific expression of PrP^C^, suggesting that axonal PrP^C^ provides trophic support necessary for Schwann cell-dependent myelination [18]. In contrast, expressing PrP^C^ exclusively in Schwann cells failed to rescue the peripheral myelin phenotype, underscoring the critical role of neuronal PrP^C^ in intercellular signaling. Although transgenic goats [19] and cattle [20] lacking PrP^C^ did not display gross phenotypes indicative of PNS myelination defects, closer examination of teased nerve-fiber preparations from PrP^C^-deficient goats revealed histopathological changes characteristic of demyelinating neuropathy similar to that seen in *Prnp*^−/−^ mice [21,22]. Together, these findings suggest that PrP^C^ plays a direct role in PNS myelin maintenance.

### 3.2. Roles of the Normal Cellular Prion Protein (PrP^C^) in Myelination

One proposed role for PrP^C^ in myelin biology involves signaling through the G-protein coupled receptor, adhesion G-protein coupled receptor 6 (ADGRG6, also known as GPR126). In the PNS, PrP^C^ interacts with ADGRG6, which is expressed on Schwann cells, to activate the cyclic AMP (cAMP)–protein kinase A (PKA) signaling pathway [23,24]. This pathway promotes Schwann cell proliferation and differentiation and activates expression of *Egr2* (also known as *Krox20*), which in turn activates transcription of myelin genes, including those encoding myelin basic protein (MBP), myelin protein zero (MPZ) and peripheral myelin protein 22 (PMP22) [23,25]. The N-terminal fragment of PrP^C^, which is generated through proteolytic cleavage, contains the binding motif for ADGRG6 and may act as the functional ligand for this pathway [23]. Whether a similar receptor–ligand mechanism operates in the CNS remains unknown, but the fact that ADGRG6 expression has not been detected in oligodendrocytes suggests that alternative receptors or signaling pathways mediate any effects of PrP^C^ on CNS myelination.

Another potential link between PrP^C^ and myelination is through metal homeostasis. The octapeptide repeat region of PrP^C^ binds copper ions and may facilitate redox reactions necessary for cellular metabolism [26,27]. In addition, PrP^C^ appears to exhibit ferrireductase activity, converting ferric iron (Fe^3+^) to its bioavailable ferrous form (Fe^2+^) [28]. This function is noteworthy because oligodendrocytes contain some of the highest levels of iron in the brain, and iron is essential for synthesizing myelin lipids, including cholesterol and sphingolipids [29,30]. Iron and copper deficiency and dysregulation have been reported in the brains of *Prnp*^−/−^ mice and patients with prion diseases such as CJD, and could result in impaired myelin synthesis or destabilization of existing myelin, thereby contributing to vacuolation and white matter degeneration [31,32].

### 3.3. Impacts of PrP^Sc^ on Myelination

The accumulation of misfolded PrP^Sc^ impairs neuronal proteostasis pathways, driving mitochondrial dysfunction and elevated oxidative stress [33,34,35,36,37,38,39]. These cellular disruptions compromise axonal metabolic support and undermine myelin sheath integrity (discussed in more detail in Section 5). In addition, PrP^Sc^ aggregates provoke a strong neuroinflammatory response, and the pro-inflammatory cytokines, reactive oxygen species (ROS), and nitric oxide released by hyper-activated glia are toxic to oligodendrocytes, further undermining myelin integrity [40,41].

### 3.4. Integrated Perspective

Taken together, the findings presented here suggest that CNS vacuolation observed in TSEs may reflect both direct, gain-of-function effects involving PrP^Sc^ toxicity, as well as indirect effects associated with loss of PrP^C^ and its normal physiological functions as it is converted into PrP^Sc^. PrP^C^ maintains iron and copper homeostasis and mediates trophic neuron-glia interactions necessary for maintaining myelin integrity through glial signaling pathways, supporting oligodendrocyte metabolism via metal homeostasis, and potentially participating in neuron–glia interactions that regulate myelin maintenance. These insights challenge the traditional view of spongiform degeneration as a purely neuronal phenomenon and instead support the hypothesis that white matter vacuolation in TSEs represents a myelination defect.

## 4. Lipid Homeostasis and Lysosomal Storage Diseases

While the composition of most cellular membranes is approximately 50% protein and 50% fat, mammalian CNS myelin is approximately 30% protein and 70% lipid [42,43]. Myelin lipids include cholesterol, phospholipids, and sphingolipids, and are essential for forming and maintaining the multilayered architecture of myelin sheaths. As a result, normal synthesis, transport and degradation of lipids by oligodendrocytes is critical to myelin health and integrity. While it is more intuitive that disrupted lipid metabolism in oligodendrocytes would affect myelin synthesis through cell autonomous mechanisms, lipid homeostasis in neurons is also important since neuronal accumulation of lipids is toxic, and axonal integrity is involved in maintaining the myelin sheath [44,45]. Within cells, lipids enter the lysosomal pathway via endocytosis or autophagy [46,47]. Lysosomal hydrolases, such as lysosomal acid lipase, break down complex lipids into free fatty acids and cholesterol, which are then trafficked to cellular membranes or the endoplasmic reticulum (ER) [48]. It is perhaps not surprising, given the critical role of lysosomes in lipid homeostasis, that lysosomal storage disorders (LSDs) are frequently associated with profound changes in myelin.

### 4.1. Myelin Lipid Synthesis

The galactolipid galactocerebroside (GalC) and its derivative, sulfatide, make up 20–30% of the total dry weight of the myelin bilayer. UDP-galactose:ceramide galactosyltransferase (also known as UDP-glycosyltransferase 8, encoded by the *UGT8* gene) catalyzes the transfer of galactose to ceramide in the final step of GalC synthesis. *Ugt8* knockout mice formed compact myelin but it contained glucocerebroside, which is not normally found in myelin, developed a tremor, showed multiple abnormalities in the CNS including hypo- and dys-myelination, intramyelin vacuolation of spinal cord fibers, and disrupted paranodal axo-glial interactions, and they died prematurely [49,50,51,52]. Exposure of spinal cords from young *Ugt8* null mice to 4-aminopyridine (4-AP), a drug that blocks potassium channels located in the paranodal region, partially rescued their action potential conduction defects [50]. Normally, these channels are occluded by the myelin sheath and do not participate in the conduction process, which suggests that abnormalities in the myelin produced in the absence of UDP-galactose:ceramide galactosyltransferase resulted in access of the drug to these potassium channels, perhaps due to paranodal abnormalities [50,52]. This ties defects in myelin lipids directly to potassium buffering, which is discussed in Section 6 of this review. Similar defects, including myelin vacuolation and paranodal junction abnormalities, were observed in mice deficient for galactose-3-O-sulfotransferase 1 (GAL3ST1, also known as cerebroside sulfotransferase, CST), which transfers the sulfate group of the sulfoglycolipids from 3′-phosphoadenosine 5′-phosphosulfate in the GalC biosynthesis pathway [53].

In addition to being a component of myelin that provides structural stability to the compacted myelin sheath, very long acyl (C22-C24) chain sphingolipids also play a role in the correct sorting and transport of MBP [54]. Loss of these sphingolipids in mice lacking ceramide synthase 2 (*Cers2*^−/−^), the enzyme that catalyzes their synthesis, resulted in myelination degeneration and detachment from axons, perhaps due to the incorporation of structurally inferior shorter-chain ceramides into the myelin sheath, as well as bilateral and symmetrical vacuolization and reactive gliosis [55].

### 4.2. Canavan Disease and N-Acetylaspartate Metabolism

Canavan disease is a well-characterized example of an LSD that links metabolic dysfunction and lipid homeostasis to white matter spongiform degeneration. It is an autosomal recessive disorder that arises from mutations in the aspartoacylase (*ASPA*) gene, which encodes an enzyme predominantly expressed in oligodendrocytes that catalyzes the hydrolysis of N-acetyl-L-aspartate (NAA) into aspartate and acetate [56,57,58,59]. NAA is synthesized in neurons and transferred to oligodendrocytes, where the resulting acetate serves as a key precursor for the synthesis of fatty acids and cholesterol required for myelin production [60,61]. Deficiency of ASPA leads to markedly elevated brain levels of NAA and a corresponding reduction in acetate availability [62,63,64]. Patients with Canavan disease develop spongiform degeneration of cerebral white matter, astrocytic swelling, and extensive demyelination [65]. Similarly, mice lacking ASPA exhibited early-onset spongiform degeneration, hypomyelination, and white matter vacuolation [66,67].

Several mechanisms have been proposed to explain how loss of ASPA function leads to white matter vacuolation [68]. Excessive accumulation of NAA may cause osmotic imbalance and lead to intracellular swelling and vacuole formation within oligodendrocytes and the myelin sheath. Deficiency of lipid precursors due to lack of acetate may impair the synthesis of essential lipids, which would disrupt myelin formation and maintenance. Elevated NAA levels might also increase oxidative stress, damaging oligodendrocytes and destabilizing myelin membranes [69]. In addition, acetate deficiency can impair histone acetylation, potentially leading to epigenetic dysregulation and altered expression of genes involved in oligodendrocyte differentiation and myelin maintenance [70]. These processes could act independently or in combination to contribute to vacuole formation.

### 4.3. PNPLA6 and Phospholipid Homeostasis

Patatin-like phospholipase domain-containing protein 6 (PNPLA6), also known as neuropathy target esterase (NTE), is a phospholipase that regulates phosphatidylcholine turnover and is essential for maintaining neuronal and myelin membrane integrity [71]. It plays a key role is deacylating lysophosphatidylcholine (LPC) and lysophosphatidylethanolamine (LPE) into non-toxic components [72,73,74], and loss of PNPLA6 leads to accumulation of LPC, which intercalates into and destabilizes lipid bilayers [75]. In mice, neuron-specific knockout of NTE led to vacuolation, neurodegeneration, and ER stress [76,77,78]. ER stress disrupts the trafficking of lipids required to maintain axons, which disrupts signaling between neurons and oligodendrocytes. Since membrane lipids directly regulate inward rectifying potassium (Kir) channels, lipid imbalance in neurons can significantly disrupt the electrochemical environment of the periaxonal space by altering potassium (K^+^) homeostasis, which is discussed in detail in Section 6 of this review. Alternatively, or in addition, toxic lipids may be extruded into the periaxonal space, where their accumulation can disrupt the tight association between the axon and the myelin sheath to cause lamellar splitting and create vacuoles.

The consequences of glial-specific deletion of *Pnpla6* in mice has not been assessed, but loss of the *Drosophila PNPLA6* ortholog, *Swiss-Cheese* (*sws*), from neurons and glia or glia alone resulted in glial wrapping defects, myelin abnormalities and axon damage, as well as the formation of multilamellar bodies believed to be secondary lysosomes in the process of digesting endosomal cargo, similar to what is observed in LSDs. This brain pathology in flies was associated with locomotor defects, mitochondrial abnormalities, endoplasmic reticulum stress, and accumulation of reactive oxygen species (ROS), lipid droplets and lysosomes [76,79,80,81,82,83]. These results indicate an essential role for PNPLA6 in neurons and glia, with loss from either cell type disrupting axon-glial interactions and resulting in myelination defects and severe CNS vacuolization. In humans, mutations in *PNPLA6* cause neurodegenerative disorders including spastic paraplegia (SPG39) and syndromes with cerebellar ataxia, underscoring the critical role of this protein and neuronal lipid homeostasis in brain health [71].

### 4.4. FIG4, VAC14, and PI(3,5)P_2_ Signaling Link Lipid Homeostasis to Lysosomal Defects

A key link between lipid dyshomeostasis, lysosomal dysfunction, and spongiform degeneration has emerged from studies of mouse genetic models with disrupted phosphoinositide signaling, particularly mutations in *Factor-Induced Gene 4* (*Fig4*), which encodes a phosphoinositide phosphatase, or *Vac14*, which encodes the VAC14 component of Phosphoinositide Kinase, FYVE-finger containing (PIKFYVE) complex. FIG4 complexes with PIKfyve (also known as FAB1) and VAC14 to cleave 5-phosphate from phosphatidylinositol 3,5-bisphosphate [PI(3,5)P_2_], a lipid essential for endo-lysosomal membrane dynamics (including lysosomal fission), vesicle trafficking, and autophagy [84,85,86,87,88]. In mice, homozygosity for loss-of-function mutations in *Fig4* or *Vac14* disrupts these processes, leading to accumulation of enlarged lysosomes and cytoplasmic vacuolation in neurons and glial cells, hypomyelination, white matter degeneration, and progressive neurodegeneration [85,89]. Although transgenic rescue studies demonstrated that restoring *Fig4* expression exclusively in neurons prevented CNS vacuolation and hypomyelination [90], subsequent studies using a *Fig4* conditional knockout mouse indicated that loss of FIG4 from neurons impaired oligodendrocyte maturation, while deleting it in oligodendrocytes resulted in a more severe de/dys-myelination phenotype [88].

Oligodendrocyte-specific deletion of *Pikfyve* resulted in a severe, early-onset phenotype, with mice exhibiting significant tremor and death at 2-weeks-of-age [88]. Histopathological analysis of their brains revealed profound hypomyelination of CNS white matter and a virtual absence of mature oligodendrocytes; it is not clear, however, whether the brains of these mice exhibited spongiform change. In the same study, *Fig4*^−/−^ and wildtype primary oligodendrocyte precursor cells were shown to have mostly normal-sized lysosomes, but differentiated *Fig4*^−/−^ oligodendrocytes showed an increase in size and number of perinuclear vesicles that expressed LAMP1 and RAB7, consistent with a late endosome/lysosome identity, and these vesicles prominently labeled with an antibody against myelin associated glycoprotein (MAG). Myelin components including MAG and proteolipid protein 1 (PLP1) have been shown to transiently accumulate on the plasma membrane of oligodendrocyte cell bodies prior to being endocytosed, followed by trafficking through the endo-lysosomal pathway to reach the myelin sheet [91,92]. Live imaging of oligodendrocytes in culture demonstrated that MAG was trafficked from the plasma membrane into the endo-lysosomal pathway in wildtype and *Fig4*^−/−^ cells, but in the latter, it accumulated in enlarged LAMP1-positive vesicles, consistent with impaired intracellular trafficking of myelin components that use this pathway to reach the myelin sheet [88]. The endo-lysosomal defects observed in FIG4-deficient cells has led to the reclassification of FIG4-related disorders in humans, including Charcot-Marie-Tooth type 4J (CMT4J) and Yunis-Varón Syndrome, as LSDs.

### 4.5. PI(3,5)P2 and Mucolipidosis Type IV

Mucolipidosis type IV (MLIV), is another LSD associated with hypomyelinating leukodystrophy and myelin vacuolization, and it has important connections to FIG4 and PI(3,5)P2. MLIV is caused by mutations in the *mucolipin-1* gene (*MCOLN1*), which encodes transient receptor potential mucolipin 1 (TRPML1), a non-selective cation channel that localizes to late endosomal and lysosomal membranes and regulates lysosomal pH and homeostasis through the transport of calcium, iron, zinc, and other cations from the lysosome to the cytosol [93,94,95,96,97,98,99,100]. Because TRPML1 interacts with and is activated by PI(3,5)P2, and FIG4 plays a key role in maintaining PI(3,5)P_2_ levels on lysosomes, TRPML1 function is normally considered dependent on FIG4 activity [101]. When TRPML1 was over-expressed, the vacuolization phenotypes seen in FIG4 and VAC14 deficient cells was partially rescued, presumably because enough TRPML1 was able to localize to lysosomes to restore calcium efflux and activate lysosomal fission [102,103,104]. TRPML1 has also been shown to regulate cellular trafficking and heavy metal homeostasis, and to function as a ROS sensor, all of which affect key pathways implicated in spongiform encephalopathy discussed in this review [97,98,100].

### 4.6. Broader Spectrum of Lysosomal and Autophagic Disorders

Beyond Canavan disease and FIG4-related conditions, several other LSDs and disorders with disrupted autophagy exhibit spongiform changes and white matter pathology. In lysosomal lipid metabolism disorders such as Krabbe disease, the accumulation of lipids within lysosomes disrupts the lipid balance necessary for myelin compaction and stability, predisposing white matter to vacuolation [105]. Autophagy regulators like TMEM106B have also been implicated in neurodegenerative diseases characterized by vacuolar changes, where impaired autophagy leads to the accumulation of damaged organelles and abnormal lipid storage, ultimately compromising oligodendrocyte function and myelin integrity [106]. In mice, deletion of the endosomal sorting complex required for transport (ESCRT)-I component *Tumor Susceptibility Gene 101* (*Tsg101*) from oligodendroglia led to rapid onset vacuolation of white matter and de/dys-myelination, associated with a significant tremor [107]. As TSG101 is required for normal trafficking of ubiquitinated receptors from early endosomes into multivesicular bodies [108], it plays critical roles in endo-lysosomal trafficking and autophagy, both of which were impaired in the oligodendrocyte-specific *Tsg101* knockout mice. Disturbances in iron metabolism may also intersect with lysosomal dysfunction, since mutations in the genes encoding ferritin autophagy receptors, such as nuclear receptor coactivator 4 (NCOA4), result in iron sequestration in ferritin clusters, potentially impairing oligodendrocyte metabolism and contributing to white matter pathology [109].

### 4.7. Mechanistic Insights

Impaired lipid homeostasis and lysosomal function have the potential to contribute to spongiform degeneration through several interconnected pathways. Lipid synthesis and recycling are important for normal myelin composition and compaction. Disruptions in these processes could lead to the formation of intramyelinic vacuoles directly due to structural instability of myelin or secondary effects of paranodal junction abnormalities on potassium buffering (discussed in detail in Section 6). Endo-lysosomal trafficking is also important for the formation of structurally normal myelin, as myelin lipids are recycled via this pathway, and it is also involved in distributing some myelin proteins, such as MAG and PLP1, to the myelin sheath. Impaired vesicular trafficking, such as that caused by altered phosphoinositide signaling, disrupts vesicular transport and results in enlarged endo-lysosomal compartments, cytoplasmic vacuolation, and reduced myelin biogenesis. Osmotic stress may arise from the buildup of metabolites such as NAA, causing cellular swelling and vacuole formation. In addition, impaired lysosomal clearance of damaged organelles increases oxidative stress through elevated reactive oxygen species, further contributing to myelin degradation (discussed in detail in Section 5). Collectively, these findings emphasize that lysosomal health is fundamental for maintaining myelin integrity. Disrupted lysosomal and autophagic pathways are increasingly recognized as primary drivers of spongiform degeneration, linking diverse LSD to white matter pathology.

## 5. Mitochondria as a Central Hub

Myelination is an energy-intensive process that requires vast amounts of mitochondrial ATP to synthesize myelin lipids and proteins. To support this high metabolic output, oligodendrocytes contain the highest levels of iron in the CNS, using iron cofactors to assemble electron transport chain complexes. Mitochondrial ATP also powers the Na^+^/K^+^ ATPases, calcium exchangers, and aquaporin channels that maintain osmotic stability across myelin sheaths. Consequently, when mitochondrial quality control fails, damaged mitochondria trigger a cascade of ATP deficiency, ROS production, lipid peroxidation, and lysosomal dysfunction that ultimately compromises myelin integrity and leads to vacuolar pathology.

### 5.1. Mitochondrial Dysfunction and Oxidative Stress

Mitochondrial disorders frequently feature CNS vacuolation, particularly in myelin-rich regions. Patients with Leigh syndrome exhibit white matter spongiform degeneration with relative preservation of neurons [110]. This syndrome can be caused by mutations in over 35 different genes that encode proteins involved in mitochondrial respiration [111]. In a mouse model for Leigh syndrome involving deletion of the gene encoding NADH:ubiquinone oxidoreductase subunit S4 (NDUFS4), an accessory subunit of mitochondrial complex I, spongiform degeneration of the CNS was accompanied by elevated reactive oxygen species (ROS) and myelin defects [112,113].

Spongiform degeneration has also been observed in mice homozygous for loss-of-function mutations in genes that play important roles in regulating oxidative stress and redox homeostasis, including *superoxide dismutase 2* (*Sod2*) and the transcription factor *nuclear factor*, *erythroid derived 2*, *like 2* (*Nfe2l2,* also known as *Nrf2*) [114,115]. SOD2 localizes specifically to mitochondria and converts toxic superoxide radicals into hydrogen peroxide [114,116]. NFE2L2 activates the antioxidant response element pathway, which acts as a defense against mitochondrial ROS. It also regulates the expression of mitochondrial biogenesis factors, including peroxisome proliferator-activated receptor gamma coactivator 1-alpha (PGC-1α) and nuclear respiratory factor 1 (NRF1) [117]. Deletion of either of these genes resulted in severe mitochondrial dysfunction characterized by oxidative damage to electron transport chain complexes (I, II, and V) and decreased ATP production.

Mice lacking the E3 ubiquitin ligase, Mahogunin Ring Finger-1 (MGRN1) or its interacting partner, Attractin (ATRN), exhibit mitochondrial dysfunction, elevated ROS, and dysmyelination in the CNS, and both develop widespread CNS vacuolation [118,119,120,121,122]. While MGRN1 and ATRN have been shown to form a complex that ubiquitinates and regulates melanocortin receptors [123], their role in mitochondrial homeostasis remains unclear. MGRN1 has been reported to ubiquitinate and stabilize mitofusin-1 oligomers [124], but it is not known whether loss of this activity underlies the mitochondrial dysfunction observed in *Mgrn1* knockout mice, and no mitochondrial role has emerged for ATRN to date.

Non-genetic disruption of mitochondria is also associated with vacuolar degeneration of the white matter, as exemplified by Toxic Spongiform Leukoencephalopathy (TLE). TLE is caused by “chasing the dragon” (inhaling heated opioid vapors) and is thought to result from oligodendroglial toxicity of compounds created when opioids such as heroin or fentanyl are heated [125,126]. TLE is associated with severe mitochondrial dysfunction in oligodendrocytes, impaired energy metabolism, demyelination, and vacuole formation [127].

### 5.2. The Mitochondria-Iron-Myelin Nexus

Since mitochondria are key sites for iron-sulfur biogenesis and heme synthesis, it is no surprise that mitochondrial dysfunction leads to reduced iron-sulfur cluster synthesis [128,129]. Cells sense this as an iron deficiency, to which they respond by inducing iron influx through the transferrin receptor and the divalent metal transporter 1 (DMT1, also known as SLC11A2) [130]. Iron accumulation leads to oxidative stress and ROS generation via the Fenton reaction, where Fe^2+^ interacts with hydrogen peroxide to generate highly reactive hydroxyl radicals that cause rapid oxidation of organic compounds [131]. Evidence from *Ndufs4* knockout mice supports impaired iron-sulfur cluster biogenesis within mitochondria as a primary driver of iron dyshomeostasis and subsequent myelin instability [112,132]. ROS can also directly damage myelin through lipid peroxidation, which destabilizes the multi-layered architecture of the sheath and causes osmotic stress by altering the lipid microenvironment, disrupting Kir4.1 inwardly rectifying potassium channels and connexin networks (discussed in Section 6).

### 5.3. Integrated Perspective

Mitochondria represent a central hub, linking multiple pathways important for myelin homeostasis. Oligodendrocytes rely on high iron levels to build electron transport chain complexes and meet the high energy demands of myelin synthesis and osmotic maintenance via ion channels. Failure of mitochondrial quality control or redox defenses leads to elevated ROS, severe ATP deficiency, and lipid peroxidation, which compromises myelin integrity and lead to white matter vacuolation.

## 6. Potassium Homeostasis

White matter vacuolation arises in conditions that specifically disrupt the coordinated ionic and metabolic interactions between neurons, oligodendrocytes, and astrocytes. Glial connexins and the potassium channel Kir4.1, expressed on oligodendrocytes, act together to maintain potassium (K^+^) homeostasis during neural activity. Disruption of this spatial buffering process leads to vacuoles in oligodendrocytes and/or the myelin sheath.

### 6.1. Potassium Buffering and Kir4.1 Channels

During neuronal firing, large quantities of potassium ions (K^+^) are released into the extracellular space. Excess extracellular K^+^ can disrupt neuronal excitability and axonal conduction unless it is rapidly cleared. Astrocytes and oligodendrocytes take up excess K^+^ and redistribute it, a process called spatial buffering. They do so through the action of inwardly rectifying potassium channels, particularly Kir4.1. This channel is abundantly expressed on glial cells surrounding nodes of Ranvier and within the myelin sheath, where it helps maintain the ionic microenvironment necessary for normal axonal function [133,134,135,136,137]. In humans, mutations in the gene encoding Kir4.1 (*KCNJ10*) are associated with EAST/SeSAME syndrome, a condition characterized by epilepsy, ataxia, sensorineural deafness, and tubulopathy [138,139]. *Kcnj10* (Kir4.1) knockout mice exhibited motor defects, tremor, and hypo/dys-myelination (especially adjacent to gray matter) and severe spongiform vacuolation of spinal cord and brain stem white matter [140].

### 6.2. The Role of Glial Gap Junctions in Potassium Homeostasis

Connexins (Cx) are integral membrane proteins that can oligomerize to form gap junctions. Gap junctions between glial cells allow direct cytoplasmic continuity for the transfer of ions, metabolites, and signaling molecules [141]. In the CNS, oligodendrocytes express connexins such as Cx32 and Cx47, while astrocytes predominantly express Cx30 and Cx43 [142]. The gap junctions they form, with Cx32 primarily pairing with Cx30 and Cx47 with Cx43, result in an extensive panglial network that is critical for spatial buffering of ions (including K^+^) and providing metabolic support [143,144]. Cx32 forms reflexive gap junctions that create a “radial shortcut” across the layers of the myelin from the innermost layer (adjacent to the axon) to the oligodendrocyte cell body. These gap junctions allow K^+^ to quickly move radially, outward to the cell body instead of through the spiral path of the myelin cytoplasm. Heterotypic coupling between oligodendrocytes and astrocytes typically involves Cx47 and Cx43 and allows for K^+^ to pass from oligodendrocytes to astrocytes, which have a higher capacity to clear K^+^ [142,144,145].

In humans, autosomal recessive mutations in the *gap junction protein*, *gamma 2* gene (*GJC2*), which encodes Cx47, cause Pelizaeus–Merzbacher-like disease 1 (PMLD1) [146,147]. This disorder is characterized by CNS hypomyelination, nystagmus (rapid, uncontrollable eye movements in one or both eyes), developmental delay, and spasticity [148,149,150,151]. Mutations in *gap junction protein*, *beta 1* (*GJB1*), which encodes Cx32, cause Charcot-Marie-Tooth disease type 1X (CMT1X), an X-linked progressive peripheral neuropathy associated with progressive distal muscle atrophy, weakness, pes cavus (high arches), bilateral foot drop, reduced reflexes, and sometimes sensory abnormalities [152,153]. The phenotype of mice lacking Cx47 (*Gjc2*^−/−^) appears to be influenced by genetic background, as one knockout showed no CNS phenotype [154] while another developed vacuoles within CNS myelin [155]. Cx32-deficient (*Gjb1* knockout) mice developed an adult-onset demyelinating peripheral neuropathy, while CNS involvement included diminished myelinated fiber and myelin volume density, but no vacuolation [156,157,158,159]. Mice lacking both Cx32 and Cx47 exhibited profound CNS de/dys-myelination, white matter vacuolation, and splitting of the myelin sheath, associated with gross tremors and tonic seizures that caused death by the time the mice were 6-weeks-old [144]. The severity of vacuolation increased under conditions of elevated neuronal activity [160], consistent with a role for impaired K^+^ buffering. Genetic interaction studies provided further evidence that Cx32, Cx47 and Kir4.1 act in a shared pathway: Cx32 (*Gjb1^−/Y^*) knockout mice that were haploinsufficient for either Cx47 (*Gjc2^+/−^*) or Kir4.1 (*Kcnj10^+/−^*) displayed no phenotype, but Cx32 null mice haploinsufficent for *both* Cx47 and Kir4.1 developed significant white matter vacuolation [160]. Vacuoles appeared to be associated with the outer aspect of myelin sheaths, making them more similar in appearance to those seen in Kir4.1 deficient mice than Cx32/Cx47 double knockouts.

Other mouse models provide further evidence that glial uncoupling can lead to the formation of vacuoles within the oligodendrocyte cell body, within myelin lamellae, or both. Cx32/Cx43 double knockout mice exhibit white matter vacuoles with no ultrastructural changes in myelin [161], while Cx47/Cx30 double knockout mice develop widespread white matter vacuolation and thin myelin sheaths [162]. Interestingly, mice with astrocyte-targeted deletion of Cx43 and global loss of Cx30 (both of which are predominantly expressed on astrocytes) also developed widespread pathology of white matter tracts, with vacuolated oligodendrocytes and intramyelinic edema [163].

### 6.3. Mechanistic Insights

Within the CNS, K^+^ homeostasis influences the electrical excitability of neurons and osmotic stability of glial cells [164]. Loss of Kir4.1 function leads to the accumulation of K^+^ in the periaxonal space, creating an osmotic gradient that leads to the influx of water, vacuole formation, and splitting of lamellae of the myelin sheath. When gap junction communications are impaired, potassium buffering is also disrupted, and K^+^ ions entering oligodendrocytes during neuronal activity cannot be redistributed to the astrocyte network or moved radially out of the myelin layers. As a result, K^+^ and metabolic waste normally transferred to astrocytes accumulate within the cell body of oligodendrocytes as well as the myelin lamellae, which disrupts the osmotic balance and eventually impairs K^+^ uptake from the periaxonal space, leading to vacuole formation and splitting of the myelin sheath. The accumulation of K^+^ also impairs neuronal membrane repolarization and can induce tonic firing and trigger seizures. Loss of Cx47 also disrupts the transfer of nutrients essential for the production of myelin, such as lactate, from astrocytes to oligodendrocytes, further destabilizing myelin. The more severe phenotype observed in Cx32 deficient mice haploinsufficient for Cx47 and Kir4.1 most likely reflects impaired K^+^ handling at multiple steps.

## 7. Direct Connections Between Spongiosis and Myelin Proteins

The two major protein constituents of mammalian CNS myelin are PLP1 and MBP, with other proteins, including 2′:3′-cyclic nucleotide-3′-phosphodiesterase (CNP), and glycoproteins such as myelin-associated glycoprotein (MAG) and myelin-oligodendrocyte glycoprotein (MOG) present at lower concentrations [165]. These proteins are necessary for proper compaction and structural integrity of the myelin sheath and if they are absent or reduced, vacuoles may form as intramyelinic fluid-filled spaces that develop when myelin layers separate.

### 7.1. Proteolipid Protein 1 (PLP1)

Mutations in the gene encoding PLP1 cause X-linked myelin disorders ranging from severe Pelizaeus–Merzbacher disease (PMD) to milder Spastic Paraplegia Type 2 (SPG2) [166,167,168]. PMD is a severe, often fatal, infantile-onset leukodystrophy characterized by nystagmus, hypotonia (low muscle tone), and severe motor and cognitive impairment due to extensive hypomyelination. At the histopathological level, PMD-associated myelin defects include myelin sheath decompaction, vacuolation, and splitting [169]. PLP1 is the main protein component of CNS myelin and is expressed as two different isoforms, *PLP1* and *DM20*, which use alternative splice donor sites in exon 3 to join the same acceptor site for exon 4 [170,171]. *DM20* is more abundant prior to the onset of myelination, at which time the *PLP1* isoform becomes the predominant form. The protein encoded by the *PLP1* transcript is 35 amino acids longer than the one encoded by *DM20*, and the relative abundance of *DM20* and *PLP1* is important for myelin stability. Interestingly, given the possible mechanisms contributing to CNS vacuolation discussed elsewhere in this review, PLP1 is not only a primary component of the myelin sheath, but the extra 35 amino acids in PLP1 (absent from DM20) encode an intracellular loop that is essential for translocation of PLP1 into mitochondria, which results in increased mitochondrial mass and reduced oxidative phosphorylation [172,173,174]. Approximately 70% of PMD-associated *PLP1* mutations involve duplication of the entire *PLP1* gene, and the resulting overexpression of PLP1 leads to its misfolding and accumulation. This overloads the ER machinery and causes ER stress and activates the unfolded protein response, disrupting myelin formation and promoting oligodendrocyte apoptosis. ER stress also increases the interaction between the ER and mitochondria, which (also) leads to mitochondrial dysfunction [175,176,177,178,179,180].

### 7.2. Transcriptional Regulation of Myelination

**Figure 3:**
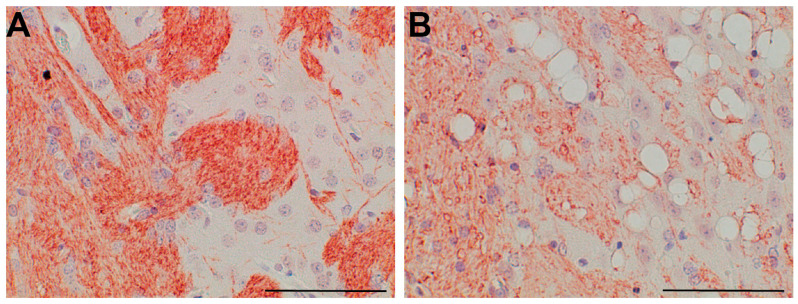
Brightfield images of immunohistochemistry for myelin basic protein (MBP, red), counterstained with hematoxylin (purple), on paraffin sections of the thalamic reticular nucleus of 3-week-old mice showing (**A**) normal and (**B**) de/dys-myelination and vacuolation in a *Sox10^gt^*^/*gt*^ mutant. Scale bars: 10 microns. Modified from Anderson et al., Mamm Genome 2014 [185] and reproduced with permission from Springer-Nature (License Number: 6311430248112).

Rare mutations in the gene encoding SRY-box transcription factor 10 (SOX10) result in central and peripheral de/dys-myelinating disease in humans (PCWH) [181,182,183,184], but it is not clear that the CNS phenotype is associated with vacuolation. In mice, however, there is evidence that disruption of SOX10 leads to spongiform encephalopathy. Specifically, mice homozygous for the *Sox10^gt^* (*gray tremor*) mutation exhibit hypo/dys-myelination of the CNS and severe vacuolation of white matter by 3 weeks of age (Figure 3), and the onset of vacuolation correlates with the initiation of CNS myelination [185,186].

The *Sox10^gt^* mutation is a point mutation that changes a highly conserved glutamic acid in the dimerization domain to glycine [185]. The dimerization domain is unique to members of the SOXE family (SOX8, 9 and 10), and promotes stable binding of SOXE hetero- or homo-dimers to DNA to activate or repress target gene expression [187,188]. It has been shown that a single intact dimerization within a SOXE dimer pair is sufficient to stabilize dimeric binding to target sequences [189], which likely explains the recessive nature of the *Sox10^gt^* mutation and suggests that SOX10 homodimers, specifically, play a critical role in OL maturation and myelination. Genes with putative SOX10 homodimer binding sites in their promoters include *PLP1*, *MBP*, *GJC2*/*CX47*, *Ugt8*, *claudin 11* (*CLDN11*), *CNP*, and *myelin regulatory factor* (*MYRF*). *PLP1*, *MBP* and *CNP* encode essential myelin components, and it is worth noting that a mutation in a SOX10 binding site in intron 3 of *PLP1* disrupts long-range regulatory interactions and affects alternative splicing to alter the balance of *DM20:PLP1*, leading to mild PMD [190,191,192]. *GJC2* encodes the Cx47 gap junction protein, connecting SOX10 to pathways discussed in Section 6 of this review. *CLDN11* encodes a critical tight junction protein that forms the radial component of myelin and acts as a diffusion barrier; when CLDN11 is absent, the myelin laminar structure is more readily disrupted by water [193,194,195]. CLDN11 deficiency causes hypomyelinating leukodystrophy (HLD22) but is not associated with vacuolation [196]. MYRF is a transcription factor that interacts with SOX10 to regulate expression of many essential myelin genes, thus playing a key role in oligodendrocyte maturation and myelin maintenance [197,198,199,200,201]. In humans, rare autosomal dominant mutations in *MYRF* cause MYRF- related mild encephalopathy with reversible myelin vacuolization (MMERV), which is associated with transient CNS myelin vacuolation [202,203]. Similarly, *Myrf* deletion in adult mouse oligodendrocytes caused severe CNS demyelination and myelin vacuolation [201].

### 7.3. Mechanistic Insights

Mutations in *SOX10* or *MYRF* disrupt the transcriptional regulation of proteins and channels, including PLP1, Cx32 and Cx47, that are essential for myelin structure and ion homeostasis. As discussed in Section 6, mutations that compromise the “tight seal” of the myelin lamellae are predicted to disrupt spatial buffering of K^+^, creating an osmotic gradient that draws water into the myelin sheath. This leads to physical separation of the lamellae intramyelinic edema or vacuoles.

## 8. Conclusions

**Figure 4:**
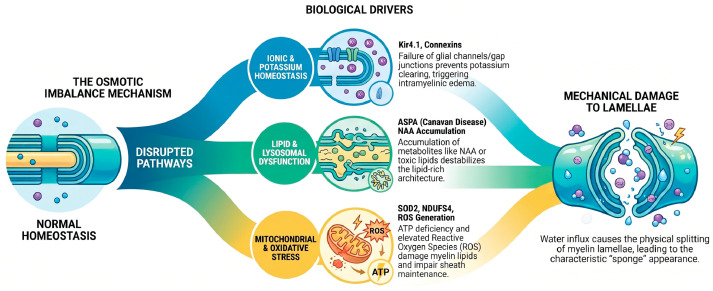
Key biological drivers of white matter vacuolation that lead to destabilization of the myelin sheath and/or osmotic imbalance. The effects may be direct, as in the case of disrupted potassium buffering or genetic mutations that impair the production of myelin lipids or proteins, or indirect, as in the case of iron dyshomeostasis or mitochondrial dysfunction, which affect myelin lipid production and stability.

Spongiform degeneration of white matter is a pathological feature observed in numerous CNS disorders. While traditionally regarded as a downstream consequence of neuronal injury, accumulating evidence suggests that white matter vacuolation reflects a structural failure in myelin. This review has presented evidence to support this hypothesis, using representative examples spanning a broad spectrum of neurological conditions, including prion diseases, lysosomal storage disorders, and mitochondrial encephalopathies, as well as mouse genetic models that exhibit spongiform encephalopathy. Taken together, these examples demonstrate that vacuolation often originates within the myelin sheath and can be driven by intrinsic oligodendrocyte defects or disrupted axon-glial communication. The diverse genetic and environmental triggers discussed here converge on a shared pathological endpoint of metabolic and ionic dyshomeostasis, which leads to osmotic swelling within the myelin lamellae to form vacuoles (Figure 4). Vacuoles, in turn, cause mechanical damage to myelin that can lead to de/dys-myelination as well as impaired axonal conduction.

Clinically, the shift in perspective proposed in this review reframes status spongiosis from an irreversible marker of neuronal loss to a dynamic, actionable target for therapeutic intervention. This is supported by the reversible nature of MMERV, which suggests that this pathology may be transient or potentially reversible if the underlying metabolic or ionic balance can be restored. Emerging gene therapy strategies and targeted molecular therapeutics are already allowing this concept to be tested with respect to inherited leukodystrophies. Results of an “n of one” clinical trial published in 2023 and a phase 1/2 clinical trial published in 2025 used recombinant adeno-associated viral vectors to drive *ASPA* expression in oligodendrocytes in Canavan disease patients [204,205]. In both reports, patients showed significant improvements, including increased white matter myelination and decreased NAA levels in cerebrospinal fluid. For PMD, strategies include: small molecular therapeutics that modulate ER stress responses or target iron-related toxicity through iron chelation; oligonucleotide therapeutics, including antisense oligonucleotides (ASOs), RNA interference (RNAi) and CRISPR-Cas9-based gene editing to reduce *PLP1* overexpression; and transplantation of wild-type glial progenitor cells [206,207]. Exciting proof-of-concept results have been observed using ASOs and CRISPR-Cas9 approaches, which significantly extended lifespan of *Plp1* mutant mice [208].

Beyond gene-specific approaches, the discovery of a shared pathological endpoint of ionic and osmotic dyshomeostasis suggests that modulating glial potassium channels could serve as a broad therapeutic strategy. Small-molecule modulators of Kir4.1 that enhance K^+^ spatial buffering are under development [209,210] and have the potential to prevent the intramyelinic edema and lamellar splitting characteristic of many vacuolar disorders. The intersection of mitochondrial dysfunction and iron dysregulation highlights the potential therapeutic benefits of antioxidant therapies or iron-sulfur cluster stabilizers to protect against the ROS-mediated lipid peroxidation that destabilizes myelin. By prioritizing the preservation of the metabolic and ionic integrity of the oligodendrocyte-axon unit, these emerging strategies offer a path toward restoring neurological function in previously untreatable conditions.

Diagnostic strategies should increasingly view white matter vacuolation as a signature of disrupted myelin formation or maintenance. Further research is needed, and this review identifies several areas of potential focus. These include dissecting the potential role of PrP^C^ in CNS myelination; elucidating the full regulatory scope of the SOX10 and MYRF network(s) to identify downstream targets; and defining how mitochondrial ROS and impaired iron-sulfur cluster biogenesis intersect with the high iron requirements of oligodendrocytes to destabilize myelin. By prioritizing the preservation of oligodendrocyte health and stabilization of the periaxonal environment, we envision the development of therapies that directly address the structural and functional failures of the CNS that disrupt myelin and lead to white matter vacuolation.

## Data Availability

No new data were created or analyzed in this study. Data sharing is not applicable to this article.

## Abbreviations

The following abbreviations are used in this manuscript:

CNS	Central nervous system
RML	Rocky Mountain Laboratory (PrP^Sc^ strain)
PNS	Peripheral nervous system
TSE	Transmissible spongiform encephalopathy
CJD	Creutzfeldt-Jakob disease
PrP^Sc^	Prion protein, scrapie form
PrP^C^	Prion protein, normal cellular form
*Prnp*	Mouse prion protein gene
ADGRG6	Adhesion G-protein coupled receptor 6
GPR126	G-protein coupled receptor 126, alternate name for ADGRG6
cAMP	Cyclic adenosine monophosphate
PKA	Protein kinase A
*Egr2*	*Early growth response 2* gene
MBP	Myelin basic protein
MPZ	Myelin protein zero
PMP22	Peripheral myelin protein 22
Fe^3+^	Ferric iron
Fe^2+^	Ferrous iron
ER	Endoplasmic reticulum
LSD	Lysosomal storage disease
GalC	Galactolipid galactocerbroside
*UGT8/**Ugt8*	UDP-galactose:ceramide galactosyltransferase gene (human/mouse)
UDP	Uridine diphosphate
4-AP	4-aminopyridine
GAL3ST1	Galactose-3-O-sulfotransferase 1
CST	Cerebroside sulfotransferase
*Cers2*	*Ceramide synthase 2* gene
*ASPA*	*Aspartoacylase* gene
ASPA	Aspartoacylase (protein)
NAA	N-acetyl-L-aspartate
PNPLA6	Patatin-like phospholipase domain-containing protein
NTE	Neuropathy target esterase
LPC	Lysophosphatidylcholine
LPE	Lysophosphatidylethanolamine
Kir	Inward rectifying potassium channels
K^+^	Potassium ion(s)
*sws*	Swiss-Cheese gene (*Drosophila*)
ROS	Reactive oxygen species
SPG39	Hereditary spastic paraplegia type 39
*Fig4/FIG4*	*Factor-induced gene 4* (mouse/human)
FIG4	Factor-induced gene 4 protein
VAC14	Vac14 homolog (S. cerevisiae) protein
PIKFYVE	Phosphoinositide kinase, FYVE-type zinc finger containing
FAB1	PIKFYVE in mammals
PI(3,5)P_2_	Phosphatidylinositol 3,5-bisphosphate
LAMP1	Lysosome-associated membrane protein 1
RAB7	RAB7, member RAS oncogene family
MAG	Myelin associated glycoprotein
PLP1	Proteolipid protein
CMT4J	Charcot-Marie-Tooth disease, type 4J
MLIV	Mucolipidosis type IV
*MCOLN1*	*Mucolipin-1* gene
TRPML1	Transient receptor potential mucolipin 1
TMEM106B	Transmembrane protein 106B
ESCRT	Endosomal sorting complex required for transport
*Tsg101*	*Tumor susceptibility gene 101* (mouse)
TSG101	Tumor susceptibility gene 101 protein
NCOA4	Nuclear receptor coactivator 4
Na^+^	Sodium ion(s)
NDUFS4	NADH:ubiquinone oxidoreductase subunit S4
NADH	Nicotinamide adenine dinucleotide
TLE	Toxic spongiform leukoencephalopathy
*Sod2*	*Superoxide dismutase 2* gene (mouse)
SOD2	Superoxide dismutase 2 protein
*Nfe2l2*	*Nuclear factor*, *erythroid derived 2*, *like 2* gene (mouse)
*Nrf2*	Alternate name for *Nfe2l2*
PGC-1a	Peroxisome proliferator-activated receptor gamma coactivator 1-alpha
NRF1	Nuclear respiratory factor 1
ATP	Adenosine triphosphate
MGRN1	Mahogunin, ring finger 1
ATRN	Attractin
*Mgrn1*	*Mahogunin*, *ring finger 1* gene (mouse)
DMT1	Divalent metal transporter 1
SLC11A2	solute carrier family 11 (proton-coupled divalent metal ion transporters), member 2
Kir4.1	inwardly rectifying potassium channel encoded by the *KCNJ10* gene
*KCNJ10*	potassium inwardly rectifying channel, subfamily J, member 10 gene (human)
*Kcnj10*	potassium inwardly rectifying channel, subfamily J, member 10 gene (mouse)
EAST	Epilepsy, Ataxia, Sensorineural deafness, and Tubulopathy
SeSAME	Seizures, Sensorineural deafness, Ataxia, Mental retardation, and Electrolyte imbalance (alternate name for EAST syndrome)
Cx	Connexin
*GJC2*	*Gap junction protein*, *gamma 2* gene (human), encodes Cx47
PMLD1	Pelizaeus–Merzbacher-like disease 1
*GJB1*	*Gap junction protein*, *beta 1* gene (human), encodes Cx32
CMT1X	Charcot-Marie-Tooth disease, type 1X
*Gjc2*	*Gap junction protein*, *gamma 2* gene (mouse)
*Gjb1*	*Gap junction protein*, *beta 1* gene (mouse)
CNP	2′:3′-cyclic nucleotide-3′-phosphodiesterase
PMD	Pelizaeus–Merzbacher disease
SPG2	Spastic paraplegia type 2
*DM20*	Diphasic myelin transcript (alternatively spliced isoform of *PLP1*)
SOX10	SRY (sex determining region Y)-box 10
PCWH	Peripheral demyelinating neuropathy, Central dysmyelinating leukodystrophy, Waardenburg syndrome, and Hirschsprung disease
*gt*	*gray tremor* (mouse *Sox10* mutant allele)
SOXE	SRY (sex determining region Y)-box family E
*Cldn11*	*Claudin-11* gene (mouse)
*Myrf*	*Myelin regulatory factor* gene (mouse)
MYRF	Myelin regulatory factor protein
HLD22	Hypomyelinating leukodystrophy
MMERV	MYRF-related mild encephalopathy with reversible myelin vacuolization
ASOs	Antisense oligonucleotids
RNAi	Ribonucleic acid (RNA) interference
